# Risk factors of renal function deterioration after radical nephroureterectomy for upper tract urothelial carcinoma

**DOI:** 10.3389/fonc.2024.1438835

**Published:** 2024-10-16

**Authors:** Qinghui Li, Tan Chen, Anli Zhu, Jie Zhou, Jiawei Zhu, Hailong Li, Rumin Wen

**Affiliations:** ^1^ Department of Urology, Luoyang Central Hospital Affiliated to Zhengzhou University, Luoyang, China; ^2^ Department of Urology, The Affiliated Hospital of Xuzhou Medical University, Xuzhou, China

**Keywords:** upper tract urothelial carcinoma, nephroureterectomy, glomerular filtration rate, renal function, acute kidney injury (AKI)

## Abstract

**Background and objectives:**

To investigate the risk factors of renal function deterioration after radical nephroureterectomy (RNU) for upper tract urothelial carcinoma (UTUC).

**Methods:**

A total of 153 patients with UTUC who underwent radical surgery at a high-volume center in China from January 2015 to December 2019 were included in this study. The renal function of all patients was evaluated during follow-up. Besides, these patients were grouped according to postoperative renal function. The risk factors of renal function deterioration included age, sex, body mass index (BMI), T stage, tumor location and size, lymph node invasion, lymph node dissection (LND), surgical margin, tumor histology, lymphovascular invasion (LVI), hypertension, diabetes, hematuria, blood transfusion, hydronephrosis on the affected side, urine specific gravity, creatinine, uric acid, and preoperative glomerular filtration rate (GFR) on the healthy and affected sides. The correlation between risk factors and inclusion indexes was analyzed using univariate and multivariate analyses.

**Results:**

A total of 153 patients were enrolled in this study, and the follow-up continued for 14 (11, 24) months. Acute kidney injury (AKI) was diagnosed in 65 patients in the short-term follow-up after RNU, and renal function deterioration was diagnosed in 52 patients in the long-term follow-up after RNU. The univariate analysis of 65 patients with short-term AKI revealed that there were statistically significant differences in preoperative hydronephrosis, hypertension, urinary protein, tumor size, preoperative Hb, preoperative creatinine, blood transfusion, and preoperative GFR of the healthy kidney. The multivariate Logistic regression analysis results showed that preoperative creatinine, GFR of the healthy kidney, and blood transfusion were independent risk factors for AKI. Moreover, The multivariate Logistic regression analysis of 52 patients with long-term renal insufficiency after surgery indicated that there were statistically significant differences in preoperative hydronephrosis, tumor size, preoperative GFR of the healthy kidney, and postoperative AKI.

**Conclusion:**

For patients with UTUC, the preoperative creatinine level is high, blood transfusion was given during or after procedure and the GFR of the healthy kidney is low, it is easy to have AKI in the short term after operation. In addition, there was no hydronephrosis before operation, the tumor size was small, the GFR of the healthy kidney was low before operation, AKI occurred after operation, the renal function was easy to deteriorate for a long time after operation. The above risk factors may aggravate renal function deterioration of these patients after surgery, resulting in the loss of the opportunity to continue treatment.

## Introduction

Urothelial carcinoma (UC) is the most common malignancy involving the urinary tract, and it can be classified into urinary bladder urothelial carcinoma (UBUC) and upper tract urothelial carcinoma (UTUC). Patients with UBUC or UTUC account for appropriately 90%-95% and 5%-10%, respectively, of all UC patients (1). Although UBUC and UTUC have similar histomorphological patterns, there are still some differences between both subtypes. From the embryological perspective, the bladder and upper urinary tract originate from two different germ cell layers. In addition, compared with UBUC, UTUC is more commonly characterized by a high histological grade, late clinical stage, and poor prognosis due to the thinner muscular layer of the upper urinary tract, more concealed location, and less severe clinical symptoms in the early stage. In the initial diagnosis, nearly 60% of UTUC was invasive, which was higher than that of UBUC (2, 3).

Low-risk diseases, defined as the presence of unifocal, small (<2 cm), low-grade, and superficial tumors, are commonly treated by kidney-sparing approaches. Radical nephroureterectomy (RNU), including the resection of the kidney, entire ureter, and bladder cuff, is the gold standard for high-risk localized UTUC (4). The results of a Phase III clinical trial of postoperative adjuvant chemotherapy in UTUC patients indicated that receiving platinum-based adjuvant chemotherapy significantly improved disease-free survival in patients with locally advanced UTUC (5). Moreover, a poor survival outcome is a primary concern among patients with locally invasive UTUC (pT2–4 or N+) and those with the necessity of perioperative chemotherapy to improve disease control. However, impaired renal functions constitute a major problem for these patients who have received RNU.

It has been demonstrated that renal functions in UTUC patients are impaired significantly after RNU. About 48%-49% of patients had an estimated glomerular filtration rate (eGFR) larger than 60ml/min/1.73m^2^ before surgery, which decreased by 19%-22% after RNU (6). On the one hand, chronic kidney disease (CKD) may be induced as there is only one kidney to fulfill the function in these patients after surgery. As revealed in previous studies, CKD may lead to worse overall survival and cancer-specific survival after the treatment of renal cell carcinoma (RCC) and UTUC (7, 8). On the other hand, Lane BR et al. (9) found that about half of the patients with a preoperative eGFR higher than 60ml/min/1.73m^2^ lost the eligibility for cisplatin chemotherapy after RNU due to renal function deterioration, which seriously affected the survival and prognosis of these patients. Therefore, it is particularly important to identify the risk factors for postoperative renal function deterioration in UTUC patients. The IDENTIFY study, an international multicenter prospective observational study, aimed to evaluate the contemporary prevalence of urinary tract cancers (including bladder cancer, upper tract urothelial cancer, and kidney cancer) in patients referred to secondary care with hematuria. Among 10,896 patients, 2,257 patients suffered from cancers (with the overall prevalence being 20.7%), and patients with bladder cancer (n = 1951) accounted for a large proportion, with the prevalence being 17.9%. Other cancer types were less common. Specifically, the prevalence of UTUC (n = 128), renal cancer, and prostate cancer was 1.17%, 0.98%, and 1.82% respectively (10). Due to the low prevalence of UTUC, the factors associated with impaired renal functions in patients with UTUC after surgery have been in a few studies.

This study was conducted to analyze the changes in renal functions and risk factors of renal function deterioration after RNU for UTUC. The results of this study may guide the perioperative management of postoperative renal function deterioration in patients with UTUC.

## Patients and methods

A total of 153 patients with UTUC who underwent radical surgery at a high-volume center in China from January 2015 to December 2019 were included in this study. The postoperative follow-up continued for more than 3 months. Only UTUC patients who had complete preoperative, postoperative, and follow-up renal function data were included in this study. The exclusion criteria included previous or concurrent radical cystectomy, contralateral or metastatic UTUC, previous renal parenchyma-sparing surgery, and preoperative systemic chemotherapy or radiotherapy. The clinicopathologic data of these patients were retrospectively collected from the medical records, including age, sex, body mass index (BMI), T stage, tumor location and size, lymph node invasion, lymph node dissection (LND), surgical margin, tumor histology, lymphovascular invasion (LVI), hypertension, diabetes, hematuria, hydronephrosis on the affected side, urine specific gravity, creatinine, uric acid, preoperative GFR on the healthy and affected sides. The GFR was evaluated according to 99mTc-DTPA. Additionally, the use of blood transfusions during and after RNU was also recorded. AKI is defined as an increase in serum creatinine of ≥0.3mg/dl or ≥1.5 times within 48 hours after surgery (11).

Firstly, the changes in the renal function of patients were analyzed based on different follow-up times. Secondly, all patients were divided into the AKI and non-AKI groups according to the renal function level within 48 hours before and after surgery. Besides, the clinicopathological characteristics of these patients in each group were evaluated. According to previous reports, the decline in renal functions was considered significant when the eGFR reduction was greater than 40% relative to baseline (12). Hence, patients with long-term renal function deterioration were grouped according to the eGFR in the last follow-up. The eGFR value was calculated through serum creatinine based on the CKD-Epidemiology Collaboration formula (13). This study was approved by the institutional review board of The Affiliated Hospital of Xuzhou Medical University. Written informed consent was obtained from all patients included in this study. To confirm the original diagnosis, an experienced urological pathologist was responsible for re-checking all pathological specimens. The tumor stage was determined based on the American Joint Committee on Cancer (AJCC) staging system. The tumor grade was defined according to the 2004 World Health Organization (WHO) classification system.

### Statistical analysis

The measurement data that did not conform to a normal distribution were expressed as the median (lower quartile, upper quartile), and the non-parametric test was used for the comparison between groups. The measurement data that conformed to a normal distribution were described by mean ± standard deviation (SD), and the T-test was used for the inter-group comparison. The statistical data were expressed as rates (%), and the χ2 test was used for the comparison between groups. The risk factors of short-term AKI and long-term renal function deterioration in UTUC patients were analyzed by the univariate analysis, and the multivariate correlation analysis was performed by a binary Logistic regression model. A two-sided p-value of <0.05 was considered statistically significant. All the statistical analyses were conducted using SPSS 26.0 (IBM Corporation, Armonk, NY, USA).

## Results

**Figure 1:**
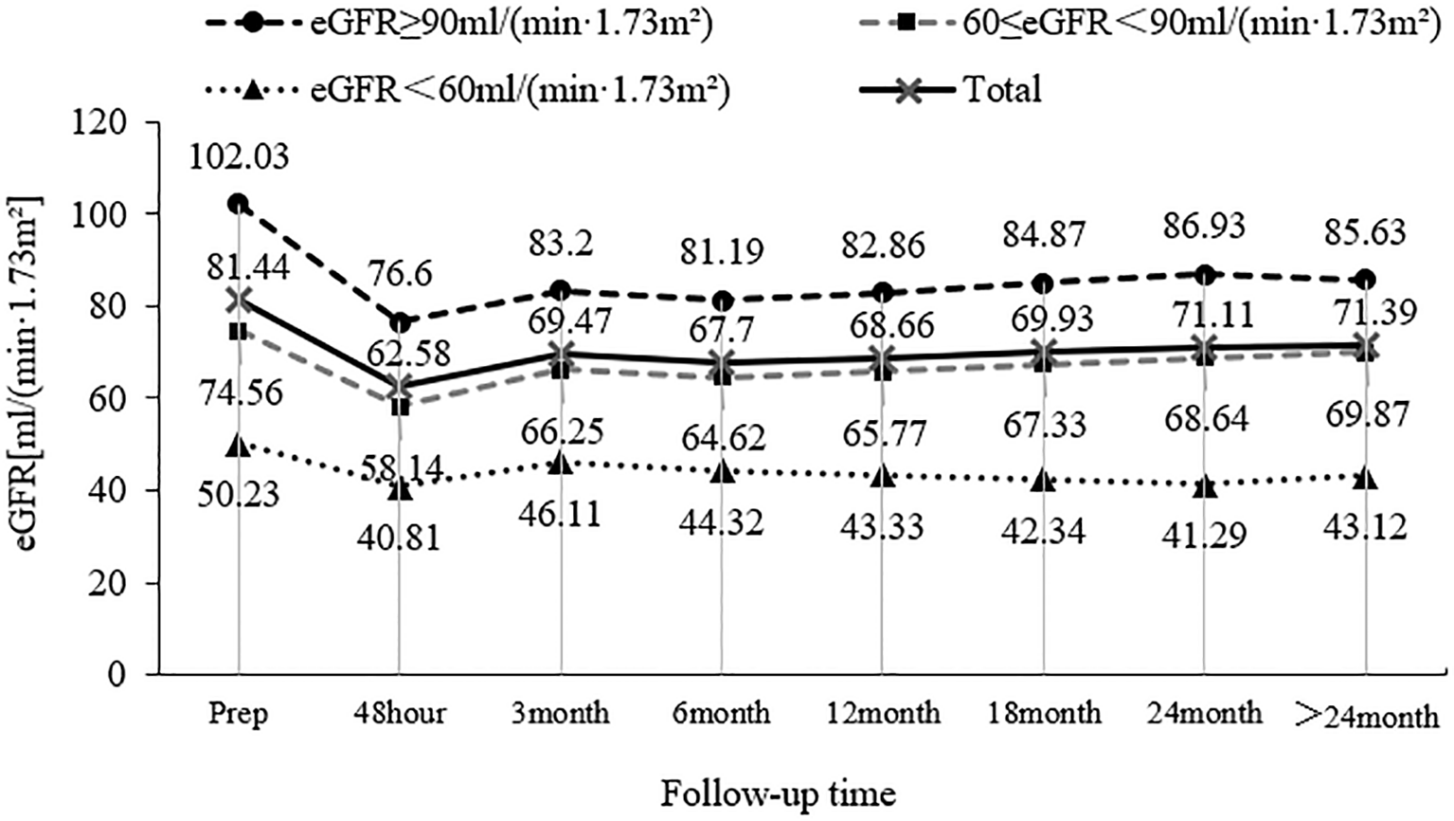
Changes in patients with different preoperative eGFR levels during follow-up.

A total of 153 patients with UTUC were included in this study, and the mean preoperative eGFR of these patients was (81.44 ± 21.30) ml/min/1.73 m^2^. Among these patients, 64 patients (41.8%) had a preoperative eGFR of ≥90 ml/min/1.73 m^2^, 60 patients (39.2%) had a preoperative eGFR between 60 and 89 ml/min/1.73 m^2^, and 29 patients (19.0%) had a preoperative eGFR of < 60 ml/min/1.73 m^2^. The mean eGFR of these patients was (62.58 ± 17.74) ml/min/1.73 m^2^ 48 hours after surgery, which was significantly different from that before surgery (P < 0.001). The mean eGFR returned to (69.47 ± 17.98) ml/min/1.73 m^2^ 3 months after surgery, exhibiting statistically significant differences compared with 48 hours after surgery (P=0.001). The overall mean eGFR remained relatively stable 6 months, 12 months, 18 months, 24 months, and > 24 months after surgery, and there was no significant difference in the eGFR at each follow-up time (P > 0.05). The changing trend of patients with different eGFR levels during follow-up before surgery is shown in **Figure 1**.

A total of 153 patients with UTUC who underwent RNU were included in this study, including 101 males and 52 females. The median age of these patients was 69 years (IQR 63-74 years). The median BMI of these patients was 23.88 (kg/m^2^) (IQR 21.94-26.08) (kg/m^2^). There were 41 patients (26.8%) with diabetes, 47 patients (30.7%) with hypertension, 102 patients (66.7%) with hydronephrosis, 81 patients (52.9%) with hematuria, and 33 patients (21.6%) requiring blood transfusions. In this study, 81 patients (52.9%) had tumors on the left side and 72 patients (47.1%) on the right side. There were 87 patients (56.8%) with the tumor located in the renal pelvis, 46 patients (30.1%) in the ureter, and 20 patients (13.1%) in both the renal pelvis and ureter. The preoperative GFR of the affected kidney was (21.00 ± 11.30) ml/min/1.73m^2^, and that of the healthy kidney was (42.54 ± 12.70) ml/min/1.73m^2^. Among the 153 patients, 65 patients (42.5%) suffered from AKI in the short-term follow-up after surgery, and 52 patients (34.0%) experienced renal function deterioration in the long-term follow-up after surgery. The clinical characteristics, laboratory data, and pathological results of all patients are shown in **Table 1**.

**Table 1:** Univariate analysis of AKI in the healthy kidney of patients after RNU (%).

Variable	AKI group (n=65)	non-AKI group (n=88)	P
Age (yr)	71 (64, 76)	68 (63, 74)	0.064
Sex			0.090
Male	38(58.5)	63 (71.6)	
Female	27 (41.5)	25 (28.4))	
BMI/kg·m^-2^	23.23 (21.47, 25.80)	24.33 (22.31, 26.67)	0.085
Hypertension			<0.001
Yes	30 (46.2)	17 (19.3)	
No	35 (53.8)	71 (80.7)	
Diabetes			0.559
Yes	19 (29.2)	22 (25.0)	
No	46 (70.8)	66 (75.0)	
Hydronephrosis Yes	34 (52.3)	68 (77.3)	0.001
No	31 (47.7)	20 (22.7)	
Hematuria			0.847
Yes	35 (53.8)	46 (52.3)	
No	30 (46.2)	42 (47.7)	
Blood transfusions			0.017
Yes	20 (30.8)	13 (14.8)	
No	45 (69.2)	75 (85.2)	
Tumor side			0.429
Left	32 (49.2)	49 (55.7)	
Right	33 (50.8)	39 (44.3)	
Tumor location			0.606
Renal pelvis	34 (52.3)	53 (60.2)	
Ureter	22 (33.9)	24 (27.3)	
Both	9 (13.8)	11 (12.5)	
pT stage			0.988
≤pT1	18 (27.7)	26 (29.5)	
pT2 pT3 pT4	16 (24.6) 25 (38.5) 6 (9.2)	22 (25.0) 33 (37.5) 7 (8.0)	
Lymph node involvement pN0 pN+ LND Yes No	49 (75.4) 16 (24.6) 21 (32.3) 44 (67.7)	76 (86.4) 12 (13.6) 18 (20.5) 70 (79.5)	0.083 0.096
Surgical margin			0.323
Positive	11 (16.9)	10 (11.4)	
Negative	54 (83.1)	78 (88.6)	
Lymphovascular invasion			0.144
Yes	20 (30.8)	18 (20.5)	
No	45 (69.2)	70 (79.5)	
Tumor histology			0.073
Papillary	19 (29.2)	15 (17.0)	
Infiltrating	46 (70.8)	73 (83.0)	
Tumor grade			0.655
High	25 (38.5)	37 (42.0)	
Low	40 (61.5)	51 (58.0)	
Tumor size/cm	3.5 (2.5, 5.1)	4.5 (3.0, 5.9)	0.019
Hb/g·L^-1^	128 ± 14	122 ± 15	0.018
Albumin/g·L^-1^	41.3 ± 4.7	42.1 ± 4.1	0.257
Creatinine/umol·L^-1^	96.0 (80.0,102.5)	69.0 (61.3,78.0)	<0.001
Uric acid/umol·L^-1^	326 (242,357)	295 (251,338)	0.343
Urine specific gravity	1.013 (1.007,1.018)	1.015 (1.011,1.019)	0.244
Urinary protein			0.013
(+)	39 (60.0)	35 (39.8)	
(–)	26 (40.0)	53 (60.2)	
Preoperative GFR on the affected side/ ml·(min·1.73m^2^) ^-1^	20.08 ± 11.57	23.37 ± 10.95	0.074
Preoperative GFR on the healthy side/ ml·(min·1.73m^2^) ^-1^	36.15 ± 11.11	48.65 ± 11.12	<0.001

A univariate analysis was performed on 153 patients with UTUC who experienced short-term AKI in the healthy kidney after RNU. The results suggested that there were no significant differences in age, sex, BMI, diabetes, hematuria, tumor side and location, T stage, lymph node involvement, LND, surgical margin, LVI, tumor histology, tumor grade, preoperative albumin, preoperative uric acid, urine specific gravity, and preoperative GFR on the affected kidney between the two groups. In addition, there was a significant difference in hypertension, hydronephrosis, blood transfusion, tumor size, preoperative Hb, preoperative creatinine, preoperative GFR of the healthy kidney, and urinary protein. The proportion of patients with preoperative hydronephrosis in the non-AKI group was higher than that in the AKI group (77.3% vs 52.3%). The proportion of patients with preoperative hypertension in the non-AKI group was lower than that in the AKI group (19.3% vs 46.2%). The proportion of patients requiring blood transfusions in the AKI group was higher than that in the non-AKI group (30.8% vs 14.8%). The tumor size of patients in the non-AKI group was larger than that in the AKI group. The 99mTc-DTPA showed that the preoperative GFR of the healthy kidney of patients in the non-AKI group was higher than that in the AKI group. The laboratory examination results showed that the preoperative Hb of patients in the non-AKI group was lower than that in the AKI group, and the preoperative creatinine of patients in the non-AKI group was lower than that in the AKI group.

To further identify the risk factors of short-term renal function deterioration on the healthy side after RNU, the statistically significant risk factors from the univariate analysis were incorporated into the multivariate Logistic risk regression model. The regression analysis results showed that there were statistically significant differences in terms of preoperative creatinine, blood transfusion, and preoperative GFR on the healthy side between the AKI group and the non-AKI group. Specifically, a higher level of preoperative creatinine and the use of blood transfusions were positively correlated with the occurrence of postoperative AKI, while a higher GFR level in the healthy kidney before surgery served as a protective factor against postoperative AKI development, as shown in **Table 2**.

**Table 2:** Multivariate analysis of AKI in the healthy kidney of patients after RNU.

Characteristic	B	OR	95%CI	P
Hydronephrosis	-0.358	0.699	0.271-1.803	0.459
Hypertension	-0.097	0.908	0.354-2.328	0.840
Tumor size	-0.211	0.810	0.653-1.004	0.055
Hb	0.030	1.030	0.998-1.063	0.065
Creatinine	0.054	1.055	1.028-1.083	<0.001
Urinary protein	0.662	1.939	0.815-4.617	0.134
Blood transfusions	1.153	3.166	1.024-9.795	0.045
Preoperative GFR on the healthy side	-0.066	0.936	0.893-0.981	0.006

B, estimated coefficient; OR, odds ratio; CI, confidence interval

Among the 153 patients enrolled in this study, a total of 52 patients exhibited renal function deterioration during long-term follow-up after surgery. A univariate analysis was conducted to investigate potential factors associated with postoperative long-term renal function deterioration. The results revealed no statistically significant differences in age, sex, BMI, hypertension, diabetes, hematuria, blood transfusion, tumor side and location, T stage, lymph node involvement, LND, surgical margin, LVI, tumor histology, tumor grade, preoperative Hb, preoperative albumin, preoperative uric acid, urinary specific gravity, urinary protein, and preoperative GFR on the affected side between the two groups. Furthermore, there were also significant differences in hydronephrosis, tumor size, preoperative creatinine, preoperative GFR of the healthy kidney, and postoperative AKI between the two groups, as shown in **Table 3**.

**Table 3:** Univariate analysis of long-term renal function deterioration in the healthy kidney after RNU (%).

Variable	Renal function deterioration group (n=52)	Non-renal function deterioration group (n=101)	P
Age/ (yr)	70 (61, 75)	69 (63, 74)	0.723
Sex			0.402
Male	32 (61.5)	69 (68.3)	
Female	20 (38.5)	32 (31.7)	
BMI/kg·m^-2^	23.89 (21.51, 26.04)	23.89 (22.05, 26.42)	0.533
Hypertension			0.136
Yes	20 (38.5)	27 (26.7)	
No	32 (61.5)	74 (73.3)	
Diabetes			0.681
Yes	15 (28.8)	26 (26.7)	
No	37 (71.2)	75 (73.3)	
Hydronephrosis			<0.001
Yes	21 (40.4)	81 (80.2)	
No	31 (59.6)	20 (19.8)	
Hematuria			0.126
Yes	32 (61.5)	49 (48.5)	
No	20 (38.5)	52 (51.5)	
Blood transfusions			0.116
Yes	15 (28.8)	18 (17.8)	
No	37 (71.2)	83 (82.2)	
Tumor side			0.387
Left	25 (48.1)	56 (55.4)	
Right	27 (51.9)	45 (44.6)	
Tumor location			0.528
Renal pelvis	29 (55.8)	58 (57.4)	
Ureter	18 (34.6)	28 (27.7)	
Both	5 (9.6 )	15 (14.9)	
pT stage			0.600
≤pT1	12 (23.1)	32 (31.7)	
pT2 pT3 pT4	13 (25.0) 21 (40.4) 6 (11.5)	25 (24.8) 37 (36.6) 7 (6.9)	
Lymph node involvement pN0 pN+ LND Yes No	39 (75.0) 13 (25.0) 17 (32.7) 35 (67.3)	86 (85.1) 15 (14.9) 22 (21.8) 79 (78.2)	0.124 0.142
Surgical margin			0.356
Positive	9 (17.3)	12 (11.9)	
Negative	43 (82.7)	89 (88.1)	
Lymphovascular invasion			0.107
Yes	17 (32.7)	21 (20.8)	
No	35 (67.3)	80 (79.2)	
Tumor histology Papillary Infiltrating	16 (30.8) 36 (69.2)	18 (17.8) 83 (82.2)	0.068
Tumor grade			0.503
High	23 (44.2)	39 (38.6)	
Low	29 (55.8)	62 (61.4)	
Tumor size/cm	3.1 (2.5, 4.8)	4.5 (3.0, 5.9)	0.002
Hb/g·L^-1^	126 ± 15	124 ± 15	0.505
Albumin/g·L^-1^	41.9 ± 3.9	41.7 ± 4.6	0.654
Creatinine/umol·L^-1^	91.0 (71.3,102.8)	69.0 (61.5,86.5)	<0.001
Uric acid/umol·L^-1^	331 (241,359)	291 (248,339)	0.160
Urine specific gravity	1.014 (1.011,1.018)	1.015 (1.011,1.019)	0.665
Urinary protein			0.282
(+)	22 (42.3)	52 (51.5)	
(–)	30 (57.7)	49 (48.5)	
AKI			<0.001
Yes No	39 (75.0) 13 (25.0)	26 (25.7) 75 (74.3)	
Preoperative GFR on the affected side/	21.54 ± 12.20	22.19 ± 10.87	0.738
ml· (min·1.73m^2^) ^-1^			
Preoperative GFR on the healthy side/ ml· (min·1.73m^2^) ^-1^	35.62 ± 11.49	47.31 ± 11.43	<0.001

To further identify the risk factors associated with renal function deterioration during long-term follow-up, the risk factors that exhibited statistically significant differences in the univariate analysis (P < 0.05) were included in a multivariate logistic regression model. The results showed that there was no statistically significant difference in preoperative creatinine between the two groups. However, significant differences in preoperative hydronephrosis, tumor size, preoperative GFR of the healthy kidney, and postoperative AKI were observed between the two groups. In addition, preoperative hydronephrosis, tumor size, and GFR of the healthy kidney were identified as protective factors against long-term renal function deterioration, as shown in **Table 4**.

**Table 4:** Multivariate analysis of long-term renal function deterioration in the healthy kidney after RNU.

Characteristic	B	OR	95%CI	P
Hydronephrosis	-1.652	0.192	0.076-0.486	0.001
Tumor size	-0.366	0.693	0.529-0.908	0.008
Creatinine	0.010	1.010	0.985-1.035	0.453
Preoperative GFR on the healthy side	-0.047	0.954	0.911-0.999	0.045
AKI	1.354	3.872	1.463-10.246	0.006

B, estimated coefficient; OR, odds ratio; CI, confidence interval

## Discussion

According to the European Association of Urology (EAU) Guidelines on Upper Urinary Tract Urothelial Carcinoma, low-risk tumors are defined as single-focal and low-grade tumors with a size of less than 2cm and without invasion on urography. Conversely, high-risk tumors encompass those with hydronephrosis or histological variations. Additionally, patients who have previously undergone radical cystectomy (RC) due to high-grade pathology are also defined to have high risks (4). Renal function deterioration is a major concern after nephroureterectomy. Patients with UTUC usually have worse baseline renal functions and overall health conditions than those with RCC (14). It has been proposed in many studies that renal functions are related to the prognosis, tumor recurrence, and survival of UTUC patients. For example, Chen CS et al. (15) found that the preoperative eGFR <30ml/min/1.73m^2^ and tumor multifocality were significant predictors of contralateral upper tract recurrence after RNU for UTUC. In addition, RNU has been recognized to exert a negative impact on renal functions and may facilitate the development of CKD (16). At the same time, as renal functions can be considered a reflection of the number of nephrons and health status, the complete resection of one kidney will impair renal functions and put patients at risk of renal failure (17). Kaag M et al. (18) found that there was a significant decline in renal functions after RNU even in patients with excellent renal reserve before surgery. Therefore, exploring the risk factors of postoperative renal function deterioration in UTUC patients has become a hot topic and received increasing attention from urologists. Consequently, this study was conducted to comprehensively examine changes in renal functions of UTUC patients after RNU and identify key factors influencing renal function deterioration.

In this study, the results revealed that the preoperative eGFR of these UTUC patients was (81.44 ± 21.30) ml/min/1.73m^2^, which subsequently decreased to (62.58 ± 17.74) ml/min/1.73m^2^ within 48 hours after surgery. However, the mean eGFR returned to (69.47 ± 17.98) ml/min/1.73m^2^ 3 months after surgery. The short-term renal function deterioration in patients with UTUC is a major impediment to adjuvant therapy in the golden treatment period after nephroureterectomy (19). In this study, the results confirmed the marked short-term renal function decline after nephroureterectomy, potentially eliminating the opportunity for adjuvant therapy. Due to the abrupt loss of a substantial number of nephrons and the failure of the healthy kidney to provide full functional compensation, a rapid decline in the eGFR was observed in the initial period after surgery. Subsequently, the remaining nephrons in the healthy kidney gradually exhibited functional compensation, characterized by compensatory hypertrophy and subsequent increases in the renal volume. As a result, the eGFR increased progressively, and renal functions recovered slowly in a short-term recovery stage. In later stages, there was no significant fluctuation in renal functions, indicating a relatively stable period. One study have been shown in adults who have undergone radical nephrectomy for the presence of a renal mass. Contralateral renal hypertrophy up to 13% was noted and did not vary based on age or gender of the patient (20). The duration of renal compensation on the healthy side has been demonstrated to be approximately 2-4 weeks in animal experiments (21). However, due to inter-individual variations and the influence of multiple other factors on renal functions in clinical practice, some patients may experience postoperative AKI with delayed recovery or non-recovery of renal functions.

The results of this study demonstrated that the preoperative GFR level in patients with healthy kidneys was an independent risk factor for both AKI and long-term renal function deterioration. In a study of postoperative renal function deterioration in patients with UTUC, the multivariate analysis results also confirmed the statistical significance of the preoperative eGFR (95%CI: 1.00-1.09, P=0.047). Further, in a multivariate logistic regression model, the pre-operative eGFR (OR 1.04; p = 0.047) was found to be an independent predictor of an eGFR reduction higher than 40% in the last clinical evaluation at a median of 15 (IQR5-30) months (22). Kaag M et al. retrospectively examined changes in the eGFR during the early (1-5 months) and late (more than 5 months) period after surgery. They corroborated that the eGFR decreased after RNU compared with that before surgery. The multivariate analysis results unraveled that the preoperative eGFR < 60ml/min/1.73m^2^ was an independent risk factor for early and late renal function deterioration after RNU (18). Similarly, Zargar H et al. delved into the influencing factors of advanced renal functions after partial nephrectomy. They found that the preoperative eGFR (P=0.0001) was a significant predictor of the degree of advanced GFR preservation (23), which was consistent with the results of this research.

It was demonstrated in this study that there was a significant association between preoperative hydronephrosis and long-term postoperative renal function deterioration, as indicated by both the univariate analysis (P < 0.001) and multivariate analysis (P=0.001), suggesting the role of preoperative hydronephrosis as a protective factor in resisting the deterioration. However, it remains undefined whether there is a relationship between hydronephrosis and postoperative renal function deterioration in UTUC patients. Tafuri A et al. (22) found that a total of 20 patients had renal function deterioration during the last follow-up, and both the univariate analysis (P=0.002) and multivariate analysis (OR=0.172, P=0.016) showed a statistically significant difference between preoperative hydronephrosis and postoperative renal function deterioration. There was an inverse correlation between hydronephrosis and outcomes of these patients, namely that, the presence of hydronephrosis implied a lower possibility of renal function deterioration after surgery. Lee BH et al. (24) conducted an exploration based on 118 UTUC patients and found that over half of the patients with a preoperative eGFR < 60 ml/min/1.73m^2^ achieved eGFR recovery within the first 3 years after RNU, and hydronephrosis was a significant predictor of recovery. Therefore, it is recommended to consider the presence of hydronephrosis in patients when discussing the progression of CKD and determining the timing of perioperative chemotherapy in high-risk individuals. This situation may be attributed to the fact that in patients with unilateral lesions and obstructive diseases before RNU, the other healthier kidney accounts for a higher proportion of the renal function, which implies that the renal function after RNU is more similar to that before RNU. Additionally, the presence of hydronephrosis enhances compensatory effects on the contralateral isolated kidney. Consequently, only a minimal loss of renal functions was observed after surgery in patients with lateral hydronephrosis. Hence, it is necessary to further explore the occurrence mechanism of functional compensation in the contralateral kidney.

The risk factors associated with renal function deterioration during long-term follow-up were also investigated in this study. The results proved a significant difference in short-term postoperative AKI in both univariate analysis (P < 0.001) and multivariate analysis (P=0.006). Tafuri A et al. (22) found that AKI was present in almost 50% of patients after RNU, and it was a strong predictor of renal function deterioration after RNU. This phenomenon may be attributed to acute endothelial injury leading to vessel shedding, compensatory glomerular hypertrophy following nephron losses, or fibrosis following AKI (25). However, to prevent AKI from progressing to CKD, the pathological and physiological mechanisms of this process still need to be further elucidated. The identification of such patients will assist in optimizing perioperative management and reducing the occurrence of postoperative AKI and its consequences. Perioperative AKI may be prevented by implementing special preoperative, intraoperative, and postoperative measures, including avoiding nephrotoxic drugs, closely monitoring serum creatinine levels and urine volume, and improving hemodynamics.

The risk factors for renal function deterioration during long-term follow-up were also explored in this study. It was found that the tumor size exhibited significant differences in both the univariate analysis (P=0.002) and multivariate analysis (P=0.008). Fang D et al. (26) investigated the risk factors for postoperative renal function deterioration in patients with renal cancer and found that the tumor size was an independent risk factor for postoperative renal function deterioration. After radical nephrectomy in patients with renal cancer, a larger tumor size was associated with a better postoperative eGFR, likely attributable to the removal of fewer healthy nephrons compared with smaller tumors (27). Zabor EC et al. (28) revealed a significant proportion of patients experiencing eGFR recovery after radical nephrectomy. The tumor size emerges as a crucial factor associated with eGFR recovery, with larger tumors resulting in renal function restoration more frequently. This could be attributed to the fact that in patients with a larger tumor size, the healthy kidney assumes a predominant role in contributing to the overall eGFR. Additionally, the healthy kidney has compensatory hypertrophy before surgery due to the larger tumor volume on the affected side. However, it remains unclear about the biological mechanism underlying contralateral renal compensation and factors influencing patient compensation, necessitating further investigations.

In this study, it was found that there was no significant difference in postoperative renal function deterioration during long-term follow-up between patients with hypertension and those with diabetes (P > 0.05). Meyer JP et al. (6) retrospectively analyzed 131 patients with RNU, with a median follow-up period of 5 years, and revealed an eGFR decline of 18% during the follow-up. The renal function deterioration was more severe in the elderly and patients with diabetes, hypertension, and previous kidney damage. However, Lee BH et al. (24) reported that there was no statistically significant difference in long-term renal function deterioration between patients with preoperative hypertension and diabetes. It has been demonstrated that prolonged dysregulation of glucose metabolism leads to glomerular ultrafiltration, dilation of the afferent arteriole, and increased renal plasma flow in patients with diabetes, ultimately contributing to progressive renal function deterioration over time (29). Similarly, Kaneko T et al. (30) corroborated that CKD and hypertension were closely correlated with each other, and hypertension induced and aggravated CKD. Chronic hypertension can result in sustained high perfusion and filtration rates within the kidneys, leading to elevated glomerular pressure and endothelial damage, which can lead to varying degrees of renal dysfunction over an extended period. However, due to a relatively short median follow-up period (14 months), these anticipated findings were not confirmed during the long-term monitoring in this study. Consequently, no statistically significant difference was observed in this study. With the extension of follow-up time, patients with hypertension and diabetes may present with changes in renal functions.

As revealed in several studies, blood transfusions or transfusion reactions may exert a potential impact on renal functions. For instance, Nuis RJ et al. (31) revealed that blood transfusion was associated with AKI after transcatheter aortic valve implantation (TAVI). Specifically, AKI occurred in 21% of the patients after TAVI. The number of blood transfusions can be employed to predict AKI. Interventions that reduce perioperative transfusions may avoid AKI, especially in anemic patients. Similarly, in a retrospective cohort of patients receiving blood product transfusions, 25% of patients experienced transfusion reactions, and these events were associated with a twofold increase in the probability of developing AKI and some of the major adverse kidney events during long-term follow-up (32). In case of increased blood loss during RNU in patients with UTUC, blood transfusions should be frequently performed during surgery to improve oxygen delivery to the kidneys and other vital organs, with the intent of preventing ischemic injury. In this study, the proportion of blood transfusions in 65 patients with postoperative AKI was close to 30.8%. Besides, blood transfusion demonstrated statistical significance in both the univariate analysis (P=0.017) and multivariate analysis (P=0.045). Pathophysiological mechanisms can explain the correlation between red blood cell transfusion and adverse reactions of blood transfusions, including impaired oxygen delivery, diminished deformability of stored red blood cells, prothrombotic effects from the increased release of procoagulant factors, and infusion-related immunosuppression. Specifically, the administration of several units of stored blood may lead to elevated circulating levels of free hemoglobin and iron, exerting nephrotoxic effects (33). Similarly, in the transfusion reaction, such inflammatory factors as interleukins and damage molecule-related products (DAMPs) are released into the blood and filtered through the glomerulus, thereby seriously affecting the metabolism of tubular cells, altering their functions, and amplifying tissue damage. Hemolysis may also occur, thus affecting the renal parenchyma (32). However, the findings of several studies indicated that various blood transfusion types (such as red blood cells, fresh frozen plasma, and platelets) exerted distinct effects on the incidence of AKI (34). Therefore, the impact of blood transfusions on renal functions needs to be further explored in subsequent studies.

Hematuria is the most common presentation of suspected urinary tract cancers. The prediction models for urinary tract cancer in patients with hematuria have been constructed (35, 36). Similarly, some researchers investigated the preoperative predictors of decreased renal functions after RNU for UTUC. Kaag M et al. (18) reported that there was no statistically significant difference in hematuria. Therefore, it is necessary to conduct further investigations based on multicenter databases. In this study, the risk factors for long-term renal function deterioration after surgery were also examined. It was found that there was no statistically significant difference in patients with preoperative proteinuria in the univariate analysis (P=0.282). Hashimoto T et al. (37) found no significant correlation between preoperative proteinuria and postoperative eGFR in their study. Microproteinuria, including positive urinary protein, has been validated to correlate with the progression of postoperative CKD (38). This discrepancy may be explained by the fact that patients with UTUC often present with hematuria before surgery, which can also result in proteinuria. Therefore, patients who have positive urinary protein levels before surgery may be prone to a misdiagnosis, leading to a lack of statistical significance in the conclusion. Additionally, the limited number of cases included in this study and the short duration of follow-up may also contribute to these findings. Hence, the relationship between proteinuria and renal function deterioration should be explored in subsequent follow-up studies.

Simultaneously, the pathological variables were incorporated to conduct a comprehensive analysis, such as T stage, lymph node invasion, LND, surgical margin, tumor histology, and LVI. Unfortunately, no affirmative outcomes were observed in relevant analyses. In a multivariate logistic regression model by Tafuri A et al., age, preoperative hydronephrosis, and preoperative eGFR, as well as coronary artery disease and non-muscle invasive tumors, exhibited statistical significance in predicting AKI on Day 1 after surgery. However, no positive results were found regarding the impact of pathological factors on renal functions, which should be explored in subsequent studies (39).

These efforts contributed to some innovative findings to some extent. For example, postoperative AKI in patients treated with RNU for UTUC was a favorable predictor of renal function deterioration during long-term follow-up. Some high-risk patients may require adjuvant chemotherapy after surgery, which may further aggravate renal function deterioration, thus losing the opportunity for further treatment. The potential risk of renal function deterioration should be thoroughly explained for UTUC patients during preoperative follow-up, thereby enabling personalized preoperative management for individuals with a high risk of kidney function deterioration. Nevertheless, there are certain limitations in this study. Firstly, this study is a single-center and retrospective study. Therefore, these conclusions should be further verified in multi-data centers. Secondly, due to poor compliance among some patients, the follow-up duration was insufficient, and some patients discontinued follow-up after achieving favorable clinical outcomes, potentially introducing selection bias. Thirdly, relevant contributing factors for renal function deterioration may be omitted in this study. Fourthly, the eGFR was calculated by adding the creatinine value into the CKD-Epidemiology Collaboration formula, which was not as accurate as the measurement value of 99mTc-DTPA. Nevertheless, considering its cost-effectiveness features, this method still holds clinical utility.

## Conclusion

For patients with UTUC, the preoperative creatinine level is high, blood transfusion was given during or after procedure and the the GFR of the healthy kidney is low, it is easy to have AKI in the short term after operation. In addition, there was no hydronephrosis before operation, the tumor size was small, the GFR of the healthy kidney was low before operation, AKI occurred after operation, the renal function was easy to deteriorate for a long time after operation. The above risk factors may aggravate renal function deterioration of these patients after surgery, resulting in the loss of the opportunity to continue treatment.

## Data Availability

The raw data supporting the conclusions of this article will be made available by the authors, without undue reservation.
